# The Use of Thiocyanate Formulations to Create Manganese Porphyrin Antioxidants That Supplement Innate Immunity

**DOI:** 10.3390/antiox11071252

**Published:** 2022-06-25

**Authors:** Brian J. Day, Elysia Min, Jie Huang, Chris Stanley

**Affiliations:** 1 Department of Medicine, National Jewish Health, Denver, CO 80206, USA; mine@njhealth.org (E.M.); huangj@njhealth.org (J.H.); 2 Harmony Consulting, San Clemente, CA 92672, USA; harmonyconsulting@live.com

**Keywords:** manganese porphyrins, thiocyanate, hypohalous acids, haloperoxidases, antioxidants, innate immunity

## Abstract

The innate immune response to infection results in inflammation and oxidative damage, creating a paradox where most anti-inflammatory and antioxidant therapies can further suppress an already inadequate immune response. We have previously reported the beneficial effects of the exogenous supplementation of innate immunity with small pseudohalide thiocyanate (^−^SCN) in a mouse model of a cystic fibrosis (CF) lung infection and inflammation. The object of this study was to evaluate the use of ^−^SCN as a counter anion for cationic manganese porphyrin (MnP) catalytic antioxidants, which could increase the parent compound’s antioxidant spectrum against hypohalous acids while supplementing innate immunity. The antioxidant activities of the parent compound were examined, as its chloride salt was compared with the ^−^SCN-anion exchanged compound, (MnP(SCN) versus MnP(Cl)). We measured the superoxide dismutase activity spectrophotometrically and performed hydrogen peroxide scavenging using oxygen and hydrogen peroxide electrodes. Peroxidase activity was measured using an amplex red assay. The inhibition of lipid peroxidation was assessed using a thiobarbituric acid reactive species (TBARS) assay. The effects of the MnP compounds on macrophage phagocytosis were assessed by flow cytometry. The abilities of the MnP(Cl) formulations to protect human bronchiolar epithelial cells against hypochlorite (HOCl) and glycine chloramine versus their MnP(SCN) formulations were assessed using a cell viability assay. We found that anions exchanging out the chloride for ^−^SCN improved the cellular bioavailability but did not adversely affect the cell viability or phagocytosis and that they switched hydrogen-peroxide scavenging from a dismutation reaction to a peroxidase reaction. In addition, the ^−^SCN formulations improved the ability of MnPs to protect human bronchiolar epithelial cells against hypochlorous acid (HOCl) and glycine chloramine toxicity. These novel types of antioxidants may be more beneficial in treating lung disease that is associated with chronic infections or acute infectious exacerbations.

## 1. Introduction

Diseases associated with recurrent or chronic infections result in tissue damage due to the prolonged inflammation and oxidative stress initiated by the innate immune system. Cystic fibrosis (CF) is characterized by chronic recurrent lung infections and progressive lung destruction, due in part to chronic lung neutrophilia [1]. Lung-recruited neutrophils release proteolytic enzymes and generate oxidants as part of the innate immune response to pathogens [2]. Myeloperoxidase (MPO) is stored in neutrophil granules that fuse with phagosomes to produce hypohalous acids that cause oxidative damage to both pathogens and the host. Oxidants play some important roles in the phagosome-mediated killing of pathogens that involve anion channel function [3,4]. The use of antioxidants in CF has resulted in only modest improvements in clinical outcomes [5,6,7]. One potential reason for this is that antioxidants counteract some of the beneficial effects of the innate immune system oxidants need to provide an adequate host defense. 

Thiocyanate (^−^SCN) is an endogenous pseudohalide anion that is abundant in bodily fluids and a natural product from plant-based diets [8]. Dietary sources that enrich ^−^SCN levels are associated with glucoside and glucosinolate-containing plants such as those found in the *Brassicaceae* family [9]. –SCN is well-tolerated and only associated with toxicity at very high plasma levels above 1 mM concentrations [10,11]. ^−^SCN is utilized by haloperoxidases, including neutrophil myeloperoxidase (MPO) and epithelial lactoperoxidase (LPO), to generate hypothiocyanous acid (HOSCN), a wide-spectrum antimicrobial agent [12]. The ^−^SCN/HOSCN redox couple exhibits selective antimicrobial toxicity. HOSCN has been shown to be more toxic to some pathogens than the host because of its ability to be metabolized by the host thioredoxin reductase, but not by the pathogen thioredoxin reductase [13]. ^−^SCN also can readily react with all hypohalous acids and their secondary products, forming HOSCN that can be readily deactivated by the host [14,15]. These properties give ^−^SCN the ability to act as an antioxidant while retaining host defense properties and could be combined with more traditional antioxidants and anti-inflammatory agents to enhance treatments for chronic infectious disease.

**Figure 1:**
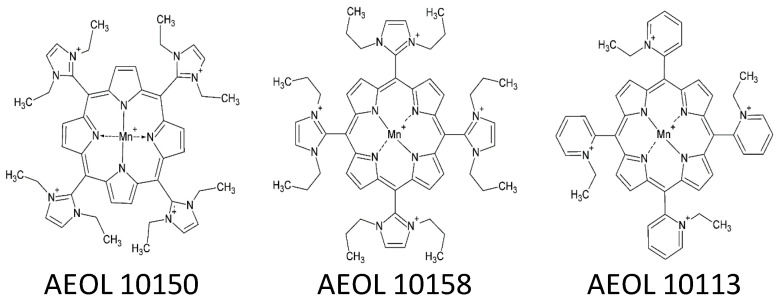
**Cationic manganese (III) *meso*-porphyrin antioxidant structures.** *Meso*-porphyrins were originally formulated as their respective chloride salts. These original chloride salt formulations were anion exchanged with thiocyanate (^−^SCN) to generate their respective ^−^SCN salt formulations.

Manganese (III) *meso*-porphyrins (MnPs) have been developed as catalytic antioxidants with the ability to perform one and two electron dismutation reactions with superoxide and hydrogen peroxide [16]. The cationic class of MnPs have a +5-charge due to the metal and the four *meso*-group N-alkyl pyridine or N, N-alkyl imidazole pendants (Figure 1). These antioxidants decrease oxidative stress and show efficacy in a number of animal models for human disease [17]. Currently, MnPs have been formulated as their chloride salts (MnP(Cl)). It was tested whether the replacement of the chloride salt with ^−^SCN (MnP(SCN)) would affect their known antioxidant activities and improve their ability to protect lung epithelial cells against hypochlorous acid (HOCl) and glycine chloramine.

## 2. Materials and Methods

**Materials and reagents:** Frozen rabbit brains were purchased from Pel-Freeze. Cytochrome c from horse heart, xanthine oxidase from bovine milk, thiobarbituric acid, L-ascorbate, ferrous chloride, glycine, sodium thiocyanate, sodium hypochlorite (10–15%), 1,1,3,3-tetramethoxypropane, cytochalasin D, and dimethyl sulfoxide were purchased from Sigma–Aldrich. Hydrogen peroxide (30%) and 1-butanol were purchased from Fisher Chemicals. DMEM and phosphate buffered saline were purchased from Corning Cellgro. Fetal calf serum was purchased from Hyclone. Amplex red, 3-(4,5-dimethylthiazol-2-yl)-2,5-dipenyl tetrazolium bromide (MTT), imaging buffer, and *Staphylococcus aureus* pH rhodo red bioparticles were purchased from Invitrogen. All reagents used were the highest reagent grade available and at least >95% purity, unless otherwise stated.

**Manganese *meso*-porphyrins anion exchange:** MnPs, their chloride (MnP(Cl)) and ^−^SCN (MnP(SCN)) salts, were provided as kind gifts by Aeolus Pharmaceuticals. The ^−^SCN content in the MnPs was determined by using HPLC with electrochemical detection (Coularray, ESA) as previously described [18]. The molar ratio of ^−^SCN to MnP was determined using MnP soret band extinction coefficients.

**Superoxide dismutase (SOD) assay:** Superoxide was generated by xanthine oxidase and detected spectrophotometrically (UV-2501PC, Shimadzu) by a reduction of cytochrome c that was assessed following the change in absorbance at 550 nm in PBS buffer at pH 7.4. A single unit of SOD activity is defined as the amount of compound needed to decrease the cytochrome c reduction rate by one half [19].

**Hydrogen peroxide assay:** Hydrogen peroxide (H_2_O_2_) consumption was measured with an electrode (WPI). The hydrogen peroxide (H_2_O_2_) electrode (World Precision Instruments, WPI) was calibrated with a 5-point hydrogen peroxide standard curve. Increasing concentrations of H_2_O_2_ were incubated with 25 μM AEOL10150 as its chloride or -SCN salt formulation at room temperature in PBS buffer at pH 7.4. H_2_O_2_ consumption was measured over 1 min using a free radical detector (WPI) and digitally recorded (Lab Scribe3). 

**Oxygen formation assay:** Oxygen formation was measured with an oxygen electrode (WPI). The oxygen probe was calibrated to a room oxygen-saturated buffer solution and oxygen formation measured in a nitrogen-purged buffer containing 1 mM H_2_O_2_ and 50 μM of AEOL10150 chloride salt formulation in the presence or absence of 1 mM NaSCN. Oxygen formation was measured over 5 min using a free radical detector (WPI) and digitally recorded (Lab Scribe 3).

**Amplex red peroxidase assay**: The assay was performed as described using the amplex red peroxidase kit microplate format with horseradish peroxidase (1 mU/mL HRP) as a positive control (Invitrogen). Reactions contained 50 μM amplex red, 1 mM H_2_O_2_, and increasing concentrations of AEOL10150(Cl). The total reaction volume was 100 μL. Reactions were incubated at room temperature in the dark for 10 min and read on a fluorescence plate reader (Synergy 2, BioTek) with an excitation of 530 nm and an emission of 590 nm. AEOL10150(Cl) alone at concentrations of up to 5 μM did not alter the background fluorescence in the assay.

**Lipid peroxidation assay:** Rabbit brain homogenates (1 mg/mL protein) were treated with iron and ascorbate for 60 min to generate hydroxyl radial-mediated lipid peroxidation in the presence of increasing concentrations of MnPs, as previously described [20]. Lipid peroxides were detected by their reaction with thiobarbituric acid and measured spectrophotometrically (SpectraxMax 340PC, Molecular Devices) at 535 nm. Non-linear regression analysis (Prism 8, GraphPad) was used to determine the 50% inhibitory concentration (IC_50_). 

**Mouse macrophage cell line (J774A.1, ATCC TIB-67)** was grown in DMEM with 10% fetal calf serum. J774A.1 cells were passed into 24 well plates and treated after reaching 90% confluence. 

**Phagocytosis Assay:** J774A.1 cells were incubated at 37 °C for 1 h in a cell image buffer (Invitrogen) with *Staphylococcus aureus* bioparticles labeled with pH-sensitive red fluoroprobe (1 mg/mL) in the presence or absence of 400 μM NaSCN, 100 μM AEOL10150(Cl), 100 μM AEOL10150(SCN), 100 μM AEOL 10113(Cl), 100 μM AEOL 10113(SCN), 100 μM AEOL 10158(Cl), 100 μM AEOL 10158(SCN), or 40 μg/mL cytochalasin D treatments. The cells were scraped and resuspended in fresh cell-imaging buffer for a flow cytometry analysis (LSR-II, BD Bioscience). The cells were gated on side scatter and PE (red) signals. Cytochalasin D-treated cells were used to set the negative gate. The total inhibition of *Staphylococcus aureus* bioparticle phagocytosis by cytochalasin D treatment was confirmed with fluorescent microscopy (EVOS_fl_, AMG). The image-buffer-only group was used to normalize the data to 100% phagocytosis and the cytochalasin D group as 0% phagocytosis. Data were analyzed using FlowJo software version 10 (TreeStar).

**Human lung bronchial epithelial cell line (16HBE41o-)** was grown in DMEM with 10% fetal-calf serum. HBE cells were passed in 24 well plates. The treatment groups contained 8 well replicates. The cells were treated with oxidant for 0.5 to 1 h in PBS and washed and replaced with fresh media. Cell viability was assessed 24 h later using a 3-(4,5-dimethylthiazol-2-yl)-2,5-dipenyl tetrazolium bromide (MTT) assay.

**Manganese porphyrin (MnP) HPLC assay:** The cellular levels of MnPs were determined by HPLC in cellular lysates as previously described with modifications [21]. Briefly, 16HBE41o- cells were grown to confluency in 6 well plates and treated with 100 μM of MnPs for 24 h. The culture media was removed and cells were washed extensively with PBS and lysed in 750 μL of PBS by brief sonication. The cellular lysates were split into two fractions; one fraction was used to determine the cellular lysate protein levels using a Coomassie protein assay (Thermo Scientific). The other fraction was deproteinated with perchloric acid (0.1 N) followed by centrifugation at 20,000× *g* for 12 min. This extraction resulted in an extraction efficiency of MnPs greater than 90%. MnP concentrations were determined by HPLC with spectrophotometric detection (Shimadzu). A 25 μL sample or standard was injected and MnPs were measured at 446 nm with retention times of 2.3 to 4.8 min. MnP concentrations were determined from standard curves that were linear over the concentrations reported.

**HOCl and glycine chloramine cytotoxicity assay:** 16HBE41o- cells were treated with 200 μM of glycine chloramine, made as previously described [22], or HOCl, for 30 min in PBS with increasing concentration of MnPs or 60 min in PBS without increasing concentrations of MnPs, respectively. Cells were washed and then placed back in media with or without MnPs, and cell viability was assessed at 24 h using the MTT assay. The percentage of protection was determined by normalizing the data to cell viability with and without oxidant. Data curves were generated using Prism 8 software (GraphPad) for 50% effective concentration (EC_50_) determinations and their respective 95% confidence intervals.

**MTT cell viability assay:** The cytotoxicity of lung epithelial cells was determined using MTT, as previously described [23]. Briefly, cells were washed with PBS and incubated in the dark at 37 °C with 0.5 mg/mL of MTT for 45 min. The reduced MTT was dissolved in DMSO and read on a plate reader at 550 nm (Synergy 2, BioTek). The absorbance was inversely correlated with cell injury.

**Statistically analysis:** Pair wise data were analyzed for statistical differences using a two-tailed t-test. Data sets with more than two treatment groups were analyzed for statistical differences using a one-way ANOVA with a Tukey’s range test for data with equal variances or a Kruskal–Wallis test with a Dunn’s range test for nonparametric data sets. Significance was established at *p* < 0.05 (Prism 9 software, GraphPad). 

## 3. Results

MnPs have been synthesized and screened for antioxidant activity over the last two decades. Several of the cationic MnPs were found to have high antioxidant activity [24] (Figure 1). Three of these cationic MnPs (AEOL10150, AEOL10158, and AEOL10113) were anion exchanged to substitute ^−^SCN for chloride. The anion-exchanged MnPs were assayed for ^−^SCN incorporation using HPLC with electrochemical detection. The compounds were found to contain on average 3.5 moles of ^−^SCN per mole of MnP. The MnP AEOL10150, in the form of its ^−^SCN salt AEOL10150(SCN), was further investigated for any changes in antioxidant properties compared with its chloride salt analog AEOL10150(Cl).

**Figure 2:**
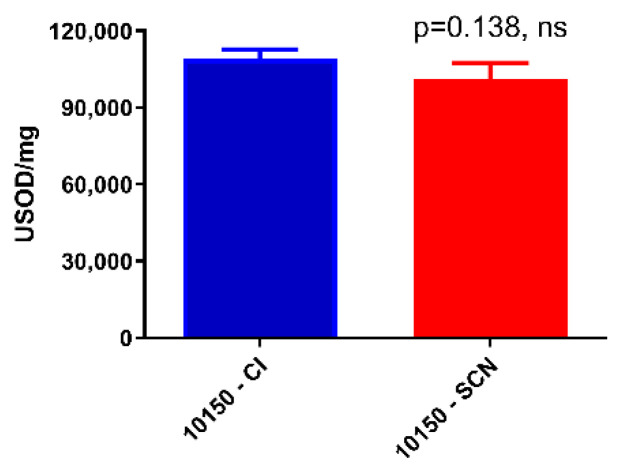
**Comparison of the superoxide dismutase (SOD) activity levels of the chloride and thiocyanate formulations of AEOL10150.** The SOD activity was determined using a cytochrome c assay spectrophotometrically. A single unit of SOD activity is defined as the ability to inhibit the rate of the reaction of superoxide with cytochrome c by one half; ns: non significant.

**Figure 3:**
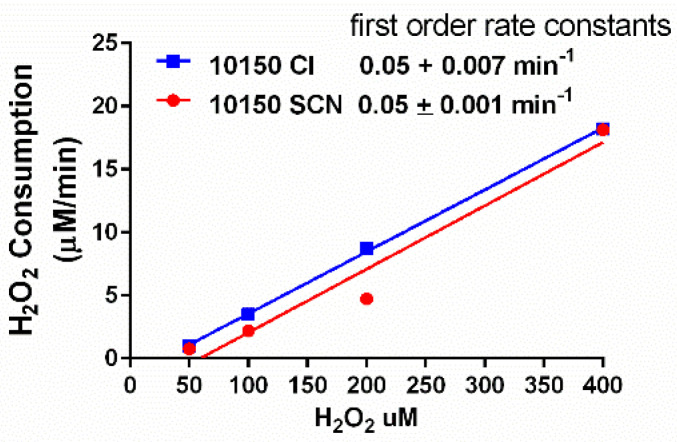
**Comparison of the hydrogen peroxide consumption of the chloride and thiocyanate salts of AEOL10150.** Samples were incubated with 25 μM of 10150 chloride (-Cl) or thiocyanate (-SCN) formulations and increasing amounts of H_2_O_2_. H_2_O_2_ consumption was determined over 1 min using a H_2_O_2_ electrode. First-order rate constants were determined from a linear regression analysis.

MnPs are broad-spectrum catalytic antioxidants known to scavenge superoxide and hydrogen peroxide, as well as to inhibit lipid peroxidation. MnPs have been reported to perform 1-electron dismutation reactions with superoxide [25]. The SOD activity of AEOL10150 was measured using a cytochrome c assay. Both salt formulations of AEOL10150 had similar SOD activities (Figure 2). MnPs also perform 2-electron dismutation reactions similar to a catalase [26]. The ability of AEOL10150 to scavenge H_2_O_2_ was measured using a H_2_O_2_-calibrated electrode, and the first order rate constants were determined for each salt formulation. Both salt formulations of AEOL10150 had similar rate constants for scavenging H_2_O_2_ (Figure 3). 

**Figure 4:**
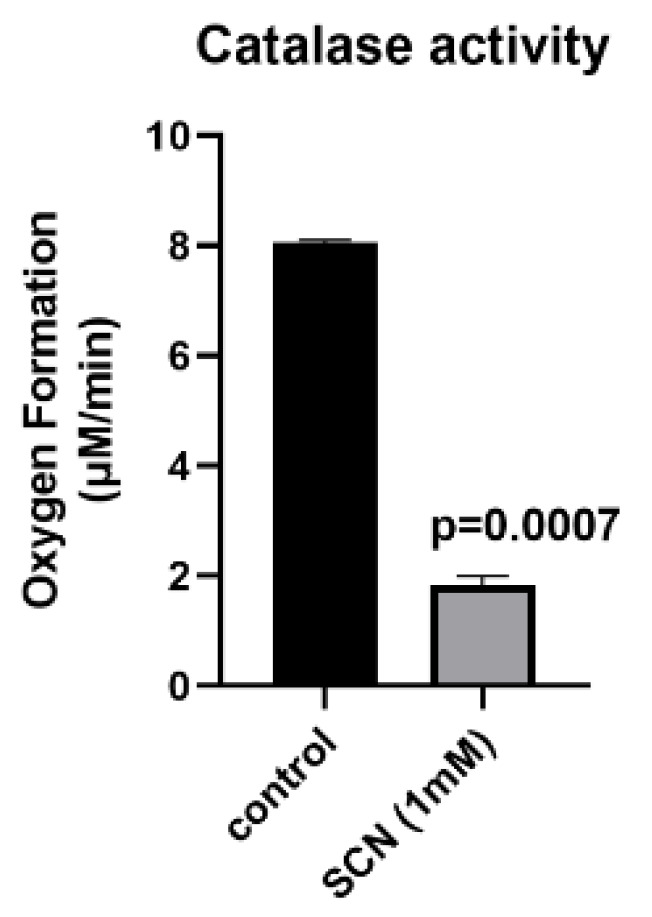
**Thiocyanate (^−^SCN) switches AEOL10150 hydrogen peroxide scavenging from a dismutation to a peroxidase reaction.** Samples were incubated with 1 mM H_2_O_2_ and 50 μM of 10150(Cl) in the presence or absence of 1 mM NaSCN, and oxygen formation was measured over 5 min using an oxygen electrode.

**Figure 5:**
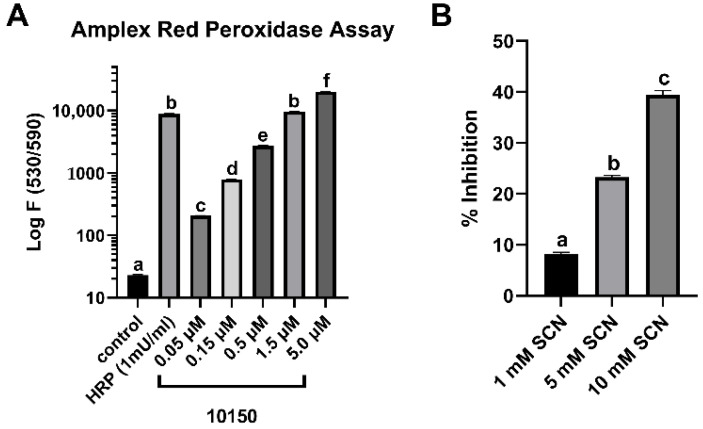
**AEOL10150 exhibits peroxidase activity towards amplex red and thiocyanate (^−^SCN)**. (**A**) Peroxidase reactions contained amplex red (50 μM) and 1 mM hydrogen peroxide (control), and fluorescence was measured after 10 min. (**B**) Peroxidase reactions contained amplex red (50 μM), hydrogen peroxide (1 mM), and 5 μM 10150, with increasing concentrations of NaSCN. The percentage of inhibition was calculated from the reactions without NaSCN. Different letters indicate a significant difference between treatment groups; *p* < 0.05.

Hydrogen peroxide can be scavenged by either a catalase-like reaction or a peroxidase reaction. The catalase dismutation of H_2_O_2_ results in the formation of oxygen and water, whereas the peroxidase reaction does not generate oxygen and instead generates water and an oxidized product. ^−^SCN is a known endogenous substrate for haloperoxidases [27]. To test whether ^−^SCN could serve as a peroxidase substrate for MnP, we assessed changes in the oxygen formation that were measured with a calibrated oxygen electrode with 1 mM hydrogen peroxide and 50 μM AEOL10150 in the absence or presence of 1 mM NaSCN (Figure 4). The addition of NaSCN most likely switched the mode of H_2_O_2_ consumption from a catalase dismutation reaction to a peroxidase reaction, as evident from the 75% inhibition of oxygen formation in the presence of 1 mM NaSCN. These data also suggest that hypothiocyanous acid (HOSCN) was formed in the reaction. The ability of AEOL10150 to function as a peroxidase was further assessed using an amplex red peroxidase assay. AEOL10150(Cl) exhibited dose-dependent peroxidase activity when compared to horseradish peroxidase (HRP) (Figure 5A). NaSCN was able to compete with amplex red as a peroxidase substrate, as evidenced by its ability to inhibit this reaction in a dose-dependent manner (Figure 5B). 

**Figure 6:**
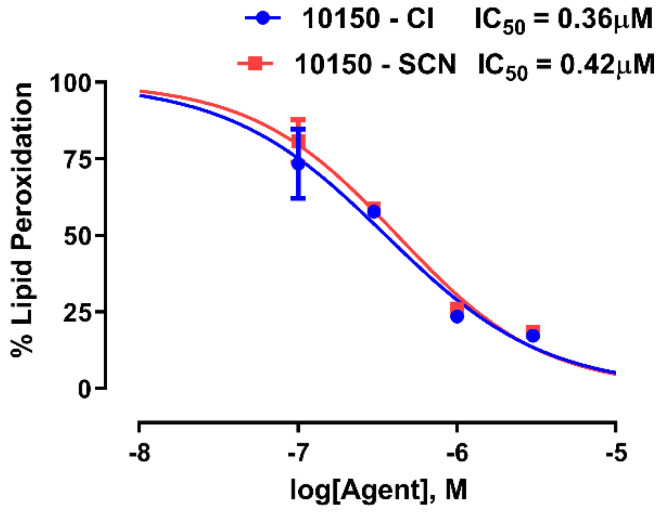
**Comparison of the lipid peroxidation inhibition activity levels of chloride (^−^Cl) and thiocyanate (^−^SCN) formulations of AEOL10150**. The lipid peroxidation of rat brain homogenates was initiated by iron/ascorbate and the lipid peroxides were measured by a thiobarbituric acid reactive species (TBAR) assay. The 50% inhibitory concentrations (IC_50_) were determined by non-linear regression.

The ability of AEOL10150 salt formulations to inhibit lipid peroxidation was determined using the iron/ascorbate-mediated lipid peroxidation of rabbit brain homogenates. The degree of lipid peroxidation was measured using a thiobarbituric acid (TBA) assay and the concentration of AEOL10150 salt formulations was determined that inhibited TBA formation by 50% (IC_50_). Both salt formulations of AEOL10150 had similar IC_50_s for the inhibition of lipid peroxidation (Figure 6).

**Figure 7:**
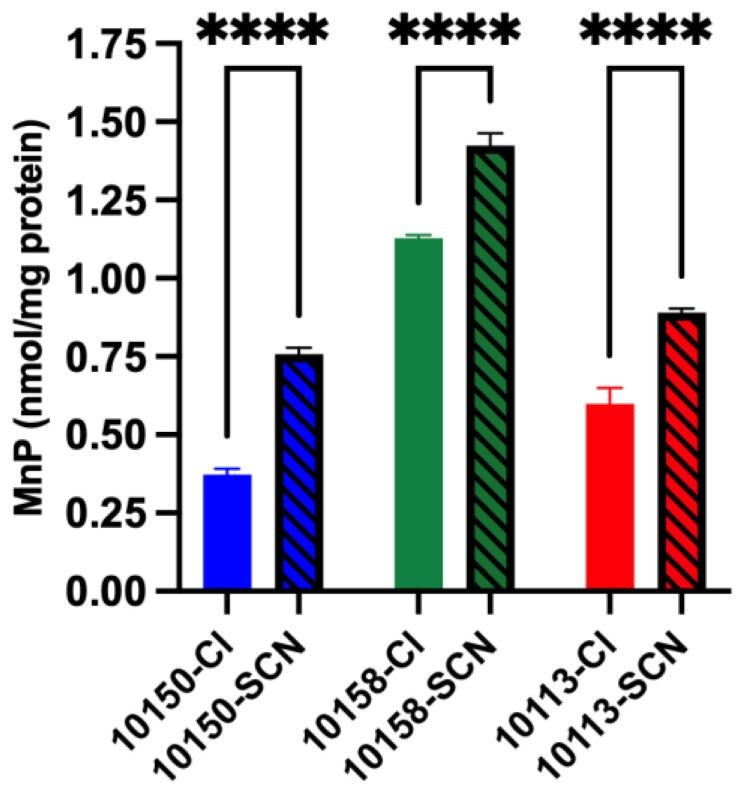
**Cellular uptake of manganese porphyrin (MnP) formulations**. Human bronchial epithelial cells (16HBE41o-) were treated for 24 h with either the MnP(Cl) or MnP(SCN) formulation and intracellular MnP concentrations were determined by HPLC. MnP(SCN) formulations had higher intracellular levels than did MnP(Cl) formulations; **** *p* < 0.001.

**Figure 8:**
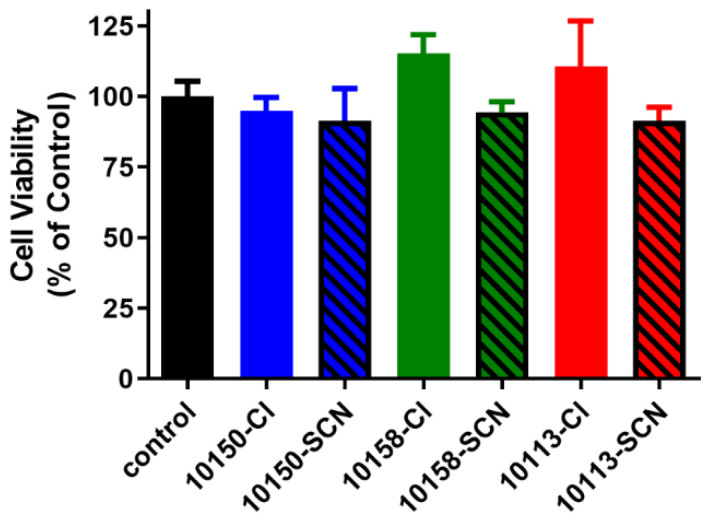
**Manganese porphyrin formulations are well tolerated by human airway epithelial cells**. Human bronchial epithelial cells (16HBE41o-) were treated with 100 μM of manganese *meso*-porphyrins (MnPs) in their chloride (Cl, solid bars) or thiocyanate (SCN, hatched bars) formulations for 24 h, and cell viability was assessed using the MTT assay. No significant differences were observed between the treatment groups and the control.

We next compared the cellular uptake of the MnPs as a function of their chloride or SCN formulation. Human bronchial epithelial cells (16HBE) were incubated with 100 μM of MnPs for 24 h. Cells were extensively washed, and lysates were made to determine the cellular levels of MnPs by HPLC (Figure 7). The MnPs in their chloride salt formulations had cellular levels that ranged from 0.37 to 1.13 nmol/mg cellular protein with AEOL10158 > AEOL10113 > AEOL10150. The MnPs in their SCN salt formulations had cellular levels that ranged from 0.76 to 1.42 nmol/mg cellular protein with AEOL10158 > AEOL10113 > AEOL10150. Changing the MnP formulation from chloride to SCN uniformly increased the cellular bioavailability by 49–79% (Figure 8).

**Figure 9:**
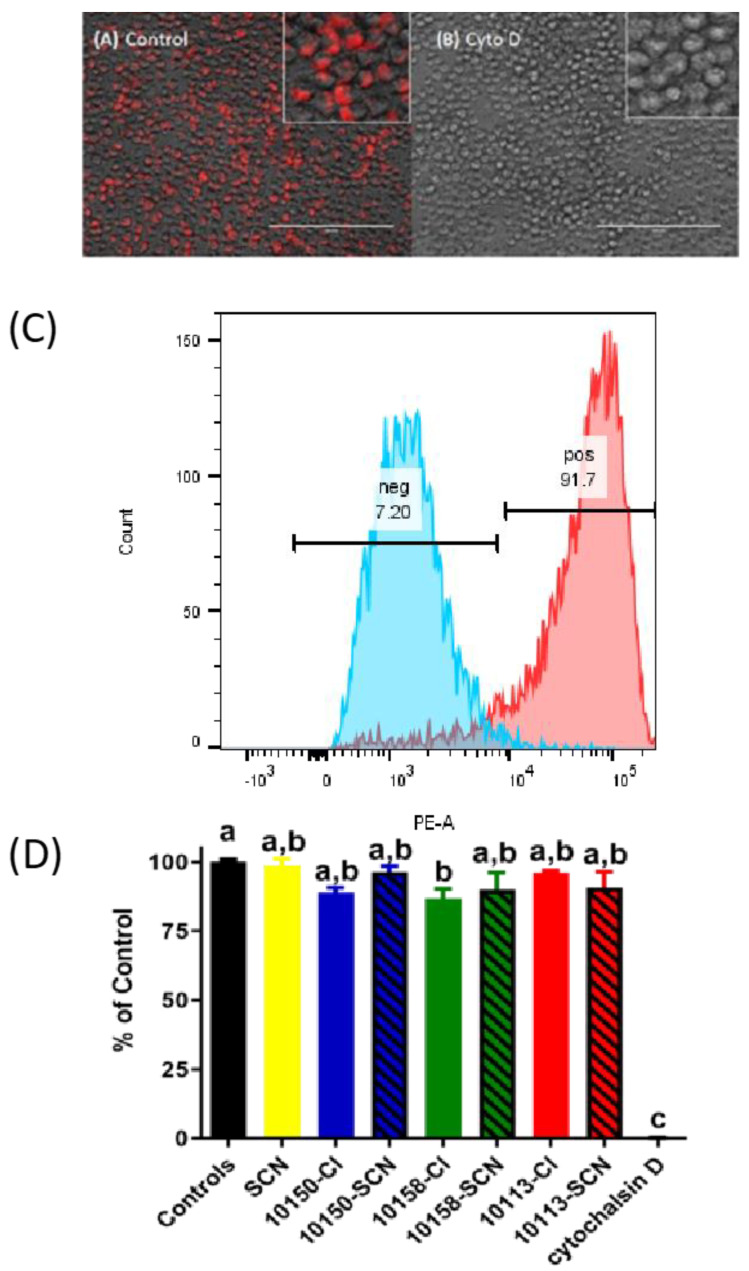
***Meso*-porphyrin formulation does not interfere with phagocytosis**. Mouse macrophage cells (J774A.1) were treated for 1 h with either 400 μM thiocyanate (SCN) or 100 μM of manganese *meso*-porphyrins (MnP) in either their chloride (Cl, solid bars) or thiocyanate (SCN, hatched bars) formulations in a cell-imaging buffer containing *Staphylococcus aureus* bioparticles labeled with pH-sensitive red fluoroprobe. The fluorescence intensity was normalized to the fluoroprobe in an imaging buffer as 100%, and the cells were treated with 40 μg/ml of cytochalasin D to inhibit the phagocytosis of the fluoroprobe as 0%. (**A**) Representative micrograph of 100% phagocytosis, and (**B**) representative micrograph of 0% phagocytosis; magnification bar is 400 μm. (**C**) A flow cytometry histogram was used to quantitate phagocytosis based on the control and cytochalasin D (40 μg/ml) positive staining cells. (**D**) Cells were gated on side scatter and PE (red) signals. Different letters indicate a significant difference between treatment groups; *p* < 0.05.

The finding that MnPs may exhibit peroxidase activity with the generation of HOSCN suggests the MnP(SCN) formulations could impact cell viability [28]. The MnP(Cl) formulations are well tolerated by lung epithelial cells up to 100 μM for 24 h. Given that the SCN formulations had higher bioavailability, we assessed cellular viability among the different formulations. Human lung bronchial epithelial cells (16HBE) were incubated for 24 h with 100 μM of each salt formulation of AEOL10150, AEOL10158, and AEOL10113. Epithelial cell cytotoxicity was assessed using a MTT cell viability assay. Both salt formulations for MnPs were equally well tolerated by the 16HBE cells (Figure 9).

We next examined whether the MnPs in their different salt formulations interfered with innate immunity. A key process of the innate immune system is the ability of the leukocytes to phagocytose pathogens. The MnPs in the form of their ^−^SCN and chloride salts were investigated for any changes in the ability of murine macrophages (J774A.1 cells) to perform phagocytosis on *Staphylococcus aureus* (SA) bioparticles. The SA bioparticles used exhibited maximum fluorescence upon acidification within the phagolysosome [29]. Flow cytometry was used to quantitate the extent of SA bioparticle phagocytosis. Cytochalasin D was used as a negative control to inhibit SA bioparticle phagocytosis and to set the negative flow cytometry gate (A and B). The inhibition of SA bioparticle phagocytosis by cytochalasin D was verified by fluorescence microscopy (Figure 9C). Only the AEOL10158(Cl) formulation had a slightly lower level of SA bioparticle phagocytosis compared to the control (Figure 9D).

**Figure 10:**
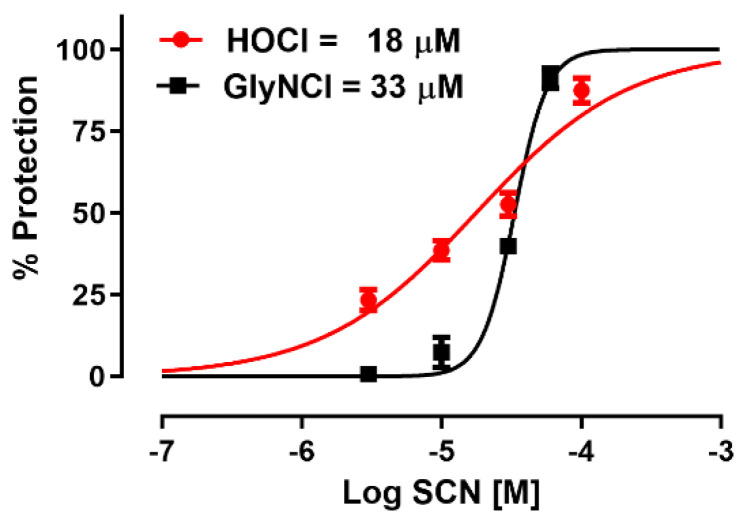
**Thiocyanate (SCN) protects human bronchial epithelial cells from hypochorous acid (HOCl) and glycine chloramine (glyNCl)-induced injury**. Human bronchial epithelial cells (16HBE41o-) were treated for 30 min with either glycine chloramine (200 μM, GlyNCl) or with HOCl (200 μM) for 60 min in PBS, then placed back in media. Cell viability was assessed 24 h later using a MTT assay. The 50% protective -SCN concentrations (EC50) were determined by non-linear regression. SCN had an EC50 of 18 and 33 μM for HOCl and GlyNCl, respectively.

**Figure 11:**
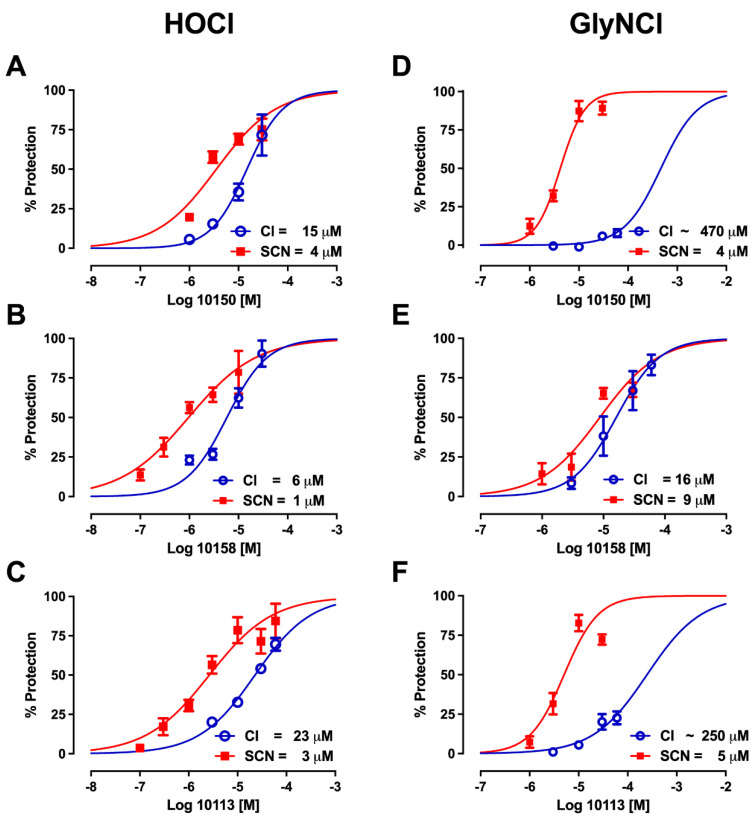
**Manganese *meso*-porphyrins protect airway epithelium from hypochlorite (HOCl) and glycine chloramine (GlyNHCl)-induced injury, and thiocyanate increases their potency**. Human bronchial epithelial cells (16HBE41o-) were treated for 30 or 60 min of either glycine chloramine (**D**–**F**) or HOCl (**A**–**C**) in PBS, then placed back in media containing manganese *meso*-porphyrins (MnPs), and cell viability was assessed 24 h later using the MTT assay. The 50% protective MnP concentrations (EC_50_) were determined by non-linear regression.

The innate immune system generates antimicrobial oxidants using haloperoxidases that are activated in phagolysomes or released extracellularly. A common oxidant generated by myeloperoxidase is hypochlorous acid (HOCl) and subsequently, chloramines as reaction by-products from the HOCl reaction with amino groups. The MnP chloride and -SCN salt formulations were tested for their ability to protect human bronchial epithelial cells against HOCl and glycine chloramine-mediated cytotoxicity. NaSCN has been previously shown to protect against these oxidants. However, MnPs have not been examined in this system before. NaSCN was first tested in this cell cytotoxicity assay against HOCl and glycine chloramine-mediated injury. NaSCN protected human bronchial epithelial cells against both HOCl and glycine chloramine-mediated injury (Figure 10). NaSCN was slightly more effective against HOCl-mediated injury, with an IC_50_ of 17.7 μM (95% CI 14–23 μM), than against glycine chloramine-mediated injury, with an IC_50_ of 33 μM (95% CI 31–36 μM). Next, the MnP(Cl) and MnP(SCN) formulations were compared for efficacy in this HOCl and glycine chloramine cytotoxicity assay. The MnP(SCN) formulations were in general more potent in protecting against both HOCl and glycine chloramine-mediated cell injury than their respective MnP(Cl) analogs (Figure 11). MnPs in their chloride formulations had protective IC_50_s that ranged from 6 to 23 μM for HOCl-mediated injury with a potency ranking of AEOL10158(Cl) > AEOL10150(Cl) > AEOL10113(Cl). MnPs as their ^−^SCN salts had protective IC_50_s that ranged from 1 to 4 μM for HOCl-mediated injury with a potency ranking of AEOL10158(SCN) > AEOL10113(SCN) > AEOL10150(SCN). MnPs as their chloride salts had variable protective IC_50_s that ranged from 16 to 500 μM for glycine chloramine-mediated injury with a potency ranking of AEOL10158(Cl) >>> AEOL10113(Cl) > AEOL10150(Cl). MnPs as their ^−^SCN salts were much more protective, with IC_50_s that ranged from 4 to 9 μM for glycine chloramine-mediated injury with a potency ranking of AEOL10150(SCN) > AEOL10113(SCN) > AEOL10158(SCN).

## 4. Discussion

The goal of this study was to combine the pseudohalide ^−^SCN, which can be utilized by the innate immune system, to generate an antimicrobial and potentially cell-signaling oxidant with an antioxidant. We demonstrate that the incorporation of ^−^SCN as a pharmacologic salt with cationic MnP antioxidants does not adversely affect the level of safety while switching the H_2_O_2_ scavenging mechanism from a dismutation reaction to a peroxidase reaction, thus mimicking the host’s haloperoxidases. In addition, the ^−^SCN salts of MnPs increased the level of protective capability against oxidants generated by the innate immune system that can damage the host and promote tissue inflammation both in vitro and in vivo. Although ^−^SCN can be protective against oxidants generated by the innate immune system, such as HOCl and chloramines, ^−^SCN lacks antioxidant activity against superoxide and H_2_O_2_ and their downstream oxidants, such as lipid peroxides. MnPs in terms of their chloride and ^−^SCN salts retain their ability to effectively scavenge superoxide and hydrogen peroxide, as well as to inhibit lipid peroxidation. It was interesting to note that the MnP mechanism for H_2_O_2_ scavenging switched in the presence of ^−^SCN from a catalase dismutation reaction to a peroxidase reaction. The peroxidase reaction would be more prominent in vivo, given that the catalase reaction operates better at high concentrations of H_2_O_2_. Endogenous ^−^SCN concentration in bodily fluids ranges from 5 to 3000 μM [12], whereas endogenous H_2_O_2_ concentration is generally in the 10–100 nM levels. The peroxidase activity of MnPs in the presence of exogenous or endogenous -SCN may support innate immune defense against pathogens by converting H_2_O_2_ to HOSCN. The reaction of MnPs with H_2_O_2_ has been shown to form relatively stable dioxomanganese (V) porphyrin complexes similar to haloperoxidases [30]. These observations suggest that MnPs may act as pseudo haloperoxidases generating HOSCN in the presence of ^−^SCN and H_2_O_2_. Given these findings, it was also interesting that the MnP(SCN) formulations were as equally well tolerated by lung epithelial cells as the MnP(Cl) formulations. MnPs have been reported to possess some antimicrobial activities [31], and this may be due to their ability to act as haloperoxidases that generate HOSCN during infection. MnP(SCN) formulations have improved our ability to protect lung epithelial cells from HOCl and chloramine-mediated injury. The improved ability is likely due to the ^−^SCN present as the counter anion. The ^−^SCN salt formulation improved MnP cellular bioavailability, which may also contribute to improving the efficacy of antioxidants. It is known that when NaSCN reacts with hypochlorous acid or chloramine, the product of this reaction generates hypothiocyanous acid [15] that is better tolerated by the host than by the pathogen [13]. This reaction provides an antimicrobial agent and potential cell-signaling oxidant [32] that may augment some of the beneficial effects of oxidants during host defense.

MnPs are effective catalytic antioxidants that efficiently scavenge superoxide and H_2_O_2_ and are efficacious in a wide variety of in vitro and in vivo models for oxidative stress [16]. However, little is known about their ability to scavenge hypohalous acids and their secondary oxidants, the haloamines. Our study showed that MnP(Cl) formulations could protect lung epithelial cells against HOCl but were much less effective in protecting against chloramines. One exception was the AEOL10158 compound, which was better at protecting lung epithelial cells against chloramines in both its chloride and ^−^SCN formulations. Some of this improved activity could be due to its cellular bioavailability, since it is better at accumulating in epithelial cells than the other two MnPs. Longer alkyl substitutions on the porphyrin pendants of AEOL10158 may be able to account for the improvement in cellular bioavailability. We observed that all the porphyrins tested showed that their ^−^SCN salt formulations had lower water solubility than their chloride salt formulations. This may be due to the lower dissociation of ^−^SCN anion from the cationic porphyrins than the chloride anion, thus making the ^−^SCN formulations more hydrophobic with better bioavailability. The increased solubility of the ^−^SCN formulations did not appear to account for all the enhanced potency of the MnPs, given that AEOL10150SCN was the most potent against glycine chloramine but the least affected by a change in cellular bioavailability. However, all three MnP(SCN)s were more effective at protecting lung epithelial cells against both hypochlorous acid and glycine chloramine than their respective chloride salts. This is likely due to ^−^SCN’s unique ability to act as a cloaked oxidant in the presence of hypohalous acids (^−^SCN/HOSCN) [12]. 

One of the features of the ^−^SCN/HOSCN system is the selective metabolism of HOSCN by the host thioredoxin reductase but not the pathogen thioredoxin reductase [13]. Another feature of HOSCN is that it is a relative selective oxidant that prefers a reaction with low pKa thiols [33]. This may impact the critical targets within pathogens that produce their known biocide effects as well as provide redox modulatory targets within the host. A potential target in the pathogen is its thioredoxin reductase which is inhibited by HOSCN [13]. Thioredoxin reductase is a critical enzyme that is needed for reducing the thioredoxin required for the ribonucteotide reductase that converts RNA to DNA [34]. HOSCN is known to form transient disulfide linkages with protein cysteine residues that readily hydrolyze to form sulfenic acids or disulfides [33,35]. This is a similar mechanism to that proposed for the H_2_O_2_-mediated cell-signaling mechanism [36,37]. In fact, it has been reported that HOSCN-treated macrophages increase the expression of cell-adhesion molecules and cytokines [32]. 

## 5. Conclusions

The use of antioxidants to treat infectious diseases associated with chronic inflammation has been disappointing, given the abundant evidence for the role of oxidative stress in these diseases [38]. For instance, both N-acetyl cysteine and glutathione have been used to treat cystic fibrosis patients with only modest changes in the clinical outcomes [5,6,7]. One of the potential limitations of using antioxidants therapeutically is the loss of the positive effect of oxidants in cell signaling and host defense. One potential approach for overcoming this limitation is to combine components of the innate immune systems with antioxidants that may reduce harm to the host while maintaining some defense against pathogens. The use of ^−^SCN as a pharmaceutical salt with MnPs may restore some of the beneficial effects that are removed during antioxidant treatments. This could only be possible if the HOSCN formed were less damaging than the other hypohalous acids. This has been demonstrated in vitro and in animal models, where inhaled or oral administration of NaSCN had positive outcomes in lung infections [18,39], ischemia-reperfusion injury [40], and atherosclerosis [41]. It will be interesting to see whether the positive effects of pairing up antioxidants with ^−^SCN in vitro hold up in more complex animal models and improve the outcomes of antioxidants in models of infectious disease and human diseases associated with chronic infections or infectious exacerbations, such as those seen in cystic fibrosis and chronic obstructive pulmonary disease.

## Data Availability

Data is contained within the article.
